# Epidemiology, treatment and outcomes of primary renal sarcomas in adult patients

**DOI:** 10.1038/s41598-024-60174-8

**Published:** 2024-05-02

**Authors:** Johannes Uhlig, Annemarie Uhlig, Hari Deshpande, Philipp Ströbel, Lutz Trojan, Joachim Lotz, Michael Hurwitz, Omeed Hafez, Peter Humphrey, Viktor Grünwald, Hyun S. Kim

**Affiliations:** 1 Department of Diagnostic and Interventional Radiology, University Medical Center Goettingen, Robert-Koch-Strasse 40, 37075 Göttingen, Germany; 2 Department of Diagnostic Radiology and Nuclear Imaging, University of Maryland School of Medicine, Baltimore, MD USA; 3 Department of Urology, University Medical Center Goettingen, Göttingen, Germany; 4 Institute of Urologic Oncology, University of California at Los Angeles, Los Angeles, CA USA; 5 Smilow Cancer Hospital, New Haven, CT USA; 6 Department of Pathology, University Medical Center Goettingen, Göttingen, Germany; 7 Department of Pathology, Yale School of Medicine, New Haven, CT USA; 8 Clinic for Medical Oncology and Clinic for Urology, University Hospital Essen, Essen, Germany

**Keywords:** Renal cancer, Renal sarcoma, Epidemiology, Survival, Surgery, Sarcoma, Cancer epidemiology

## Abstract

To assess epidemiology, clinical presentation, treatment and overall survival of adult patients with renal sarcomas, the 2004–2016 SEER and NCDB databases were queried for adult patients diagnosed with renal sarcoma, calculating average annual age-adjusted incidence rates (AAIR) and average annual percentage change (AAPC) as well as overall survival (OS). In n = 1279 included renal sarcoma patients, AAIR remained constant over the study period (average 0.53 cases/1million; AAPC = 0.7, *p* = 0.6). Leiomyosarcoma (AAIR 0.14 cases/1 million) and malignant rhabdoid tumors (0.06 cases/1 million) were most common. Sarcoma histiotypes demonstrated considerable heterogeneity regarding demographic and cancer-related variables. Patients presented with advanced local extent (T3 33.3%; T4 14.2%) or distant metastases (29.1%) and commonly underwent surgical resection (81.6%). Longer OS was independently associated with younger age, female sex, lower comorbidity index, low T stage, negative surgical margins, absence of tumor necrosis or distant metastases and leiomyosarcoma histiotype (multivariable *p* < 0.05 each). Treatment efficacy varied according to sarcoma histiotype (interaction *p* < 0.001). Accounting for 0.25% of renal malignancies, renal sarcomas include 43 histiotypes with distinct epidemiology, clinical presentation, outcomes and sensitivity to systemic therapy, thereby reflecting soft-tissue sarcoma behavior. Renal sarcoma treatment patterns follow recommendations by renal cancer guidelines with surgical resection as the cornerstone of therapy.

## Introduction

In 2018, renal cancer accounted for 2.2% of incident malignant diseases and 1.8% of cancer-related deaths worldwide^1^. The incidence of renal malignancies has increased over the last decade at an annual rate of approximately 2%, which has been attributed to an improved detection of early-stage disease through widespread use and technical advancement of cross-sectional imaging^2–4^.

While the most frequent histological subtypes of renal malignancies, including clear cell, papillary and chromophobe renal cell carcinoma (RCC), have been well studied, there is scarce literature on less frequent renal malignancies, such as primary renal sarcomas.

The term “sarcoma” refers to a heterogenous group of tumors of mesenchymal origin that can manifest throughout the body, often referred to as “soft tissue sarcomas (STS)”^5^. This heterogeneity is further reflected by the more than 100 histological and molecular sarcoma subtypes (histiotypes) with unique clinical presentation and behavior^6^.

In pediatric and adolescent patients presenting with renal masses, renal sarcomas are often considered as a differential diagnosis (e.g. nephroblastoma versus clear cell sarcoma), whereas renal sarcomas in adult patients are less common and until now have only described in smaller case series and pictorial essays^7–11^.

To date, the literature lacks a systematic evaluation of renal sarcoma epidemiology, treatment and associated outcomes on a national level with sufficient sample size to appreciate their heterogenous presentation and various histiotypes. Moreover, many renal sarcoma studies were published more than a decade ago and might not reflect the most recent advances in renal cancer imaging and treatment.

This study, therefore, aims to systematically assess epidemiology, treatment and outcomes of renal sarcomas in adult patients.

## Material and methods

Data from the US-based National Cancer Database (NCDB) and the Surveillance, Epidemiology, and End Results (SEER) program were evaluated in this study.

The NCDB, which was established in 1989 by the Commission on Cancer (CoC) of the American College of Surgeons and the American Cancer Society as a joint quality improvement project, contains data on more than 34 million cancer patients, which were recorded from over 1,500 CoC-accredited facilities across the United States. It is estimated that approximately 70% of the annually diagnosed US cancer cases are reported in the NCDB^12^. In contrast, the SEER database includes cancer cases from selected population-based state cancer registries in the United States^13^. The SEER 21 database evaluated in this study contains data from 21 cancer registries in 19 geographic areas, allowing analyses which are generalizable to the entire US population.

This study received prior approval by the local institutional review board and is Health Insurance Portability and Accountability Act (HIPAA) compliant. Informed patient consent was waived due to the study´s design evaluating anonymized databases.

### Study cohort

The 2019 NCDB participant user file containing cancer cases diagnosed between 2004 and 2016 was queried for adult patients with histopathological diagnosis of invasive (ICD-03 behavior code /3) sarcomas with renal origin (primary site codes C649, C659; ICD-03 codes provided in supplemental Table 1). Exclusion criteria were age < 18 years, missing histopathological assessment, and non-invasive behavior (ICD-03 behavior codes /1 and /2).

**Table 1:** Baseline characteristics of included NCDB patients.

Parameter	Level	Total	Male	Female	*p* value
n		1279	682 (53.3%)	597 (46.7%)	
Age					0.134
	Median (IQR)	60 (48–71)	60 (49–71)	60 (47–70)	
Race					< *0.01*
	Caucasian	1084 (84.8%)	606 (88.9%)	478 (80.1%)	
	African American	133 (10.4%)	49 (7.2%)	84 (14.1%)	
	Others	62 (4.8%)	27 (4.0%)	35 (5.9%)	
Comorbidities (Charlson Deyo Comorbidity Index)					0.62
	0	989 (77.3%)	525 (77.0%)	464 (77.7%)	
	1	208 (16.3%)	115 (16.9%)	93 (15.6%)	
	2	61 (4.8%)	29 (4.3%)	32 (5.4%)	
	> = 3	21 (1.6%)	13 (1.9%)	8 (1.3%)	
Cancer diameter [mm]					0.039
	Median (IQR)	101 (62–150)	110 (64–150)	98 (60.2–140)	
T stage					0.17
	T1	229 (17.9%)	108 (15.8%)	121 (20.3%)	
	T2	300 (23.5%)	154 (22.6%)	146 (24.5%)	
	T3	426 (33.3%)	238 (34.9%)	188 (31.5%)	
	T4	181 (14.2%)	104 (15.2%)	77 (12.9%)	
	TX	143 (11.2%)	78 (11.4%)	65 (10.9%)	
Histological tumor necrosis					*0.01*
	No necrosis	1097 (85.8%)	567 (83.1%)	530 (88.8%)	
	Necrosis	182 (14.2%)	115 (16.9%)	67 (11.2%)	
Synchronous tumor metastases					< *0.01*
	No metastases reported	867 (67.8%)	428 (62.8%)	439 (73.5%)	
	Distant metastases	412 (32.2%)	254 (37.2%)	158 (26.5%)	
Laterality					0.47
	Right	608 (47.9%)	334 (49.4%)	274 (46.1%)	
	Left	657 (51.7%)	339 (50.1%)	318 (53.5%)	
	Bilateral	5 (0.4%)	3 (0.4%)	2 (0.3%)	

Inclusion criteria for the SEER 21 database were age >  = 18 years and renal sarcoma diagnosis (as detailed above) between 2004 and 2016 to match the study period to that of the NCDB.

For exploratory analyses, incidence rates of renal sarcomas were compared to clear cell RCC (ICD-03 codes 8310 and 8312), papillary RCC (ICD-03 code 8260), and chromophobe RCC (ICD-03 code 8317).

### Variables

The SEER 21 database was used for calculation of age-adjusted incidence rates (AAIR), which were standardized to the 2000 US population. All other variables were based on NCDB data.

Renal sarcoma histiotypes were categorized into “tumor groups” according to their cellular origin as summarized in figure appendix Table 1. Further details on NCDB variables evaluated in this study are provided in the supplement.

All treatment variables provided by the NCDB referred to primary treatment of renal sarcomas. Information on therapy for recurrent sarcomas was not provided by the NCDB.

Surgical treatment of renal sarcoma was categorized as “local tumor destruction” (including thermal ablation, cryosurgery, and laser excision), “partial nephrectomy”, “radical nephrectomy”, “extended nephrectomy” (defined as radical nephrectomy and resection of other organs), “surgery, not otherwise specified (NOS)”, and “no surgery”. Surgical codes “26” (polypectomy) and “27” (excisional biopsy) were not considered to be surgical treatment. For statistical analyses, surgical treatment was dichotomized as “any surgical treatment” and “no surgical treatment”.

### Statistical analyses

Continuous variables were provided as median with inter-quartile range (IQR). Categorical variables were provided as number and frequency. Across patient subgroups, continuous variables were assessed using the non-parametric Wicoxon rank sum test, and categorical data using the chi-square test.

Temporal AAIR trends were visualized using generalized additive models.

Overall survival was assessed weighted Cox regression models with robust variance estimators due to apparent violation of the proportional hazards assumption with crossing Kaplan–Meier curves^14^. Variables were considered for inclusion in the final multivariable model if p < 0.1 in univariate analyses.

Given the variety of sarcoma histiotypes, often containing less than 10 cases, only the 8 most common histiotypes were considered as distinct strata for statistical modelling.

Age-adjusted incidence rates were calculated as annual AAIR or average annual AAIR using the SEER*Stat software. Average annual percentage change (AAPC) of AAIR were assessed using the Jointpoint regression program version 4.8.0.1.

All other statistical analyses were conducted using R version 3.6.0 (Vienna, Austria) and RStudio version 1.3.959 (RStudio Inc, Boston, MA, USA). A *p*-value of < 0.05 was defined to indicate statistical significance. All provided p-values are two-sided.

## Results

A total n = 609 renal sarcoma cases were included from the SEER 21 database and used for assessment of epidemiological trends.

Another n = 1279 renal sarcoma cases were included from the NCDB database and used for evaluation of clinical presentation, treatment, and outcomes, as detailed below.

### Epidemiology

Renal sarcoma diagnosis comprised 0.25% of all SEER-reported renal malignancies between 2004 and 2016.

There was no statistically significant change in renal sarcoma annual AAIR from 2004 to 2016 with the annual AAIR varying between 0.28 and 0.57 cases/1million and an AAPC = 0.7% (*p* = 0.6). In comparison, the annual AAIR increased for ccRCC (from 2004 to 2016: 131 to 150 annual cases/1million; AAPC = 0.9%, *p* < 0.001), papillary RCC (15 to 26 cases/1million; AAPC = 3.7%, *p* < 0.001) and chromophobe RCC (7 to 11.7 annual cases/1million; AAPC = 3.6%, *p* < 0.001; see supplemental Fig. 1).

**Figure 1:**
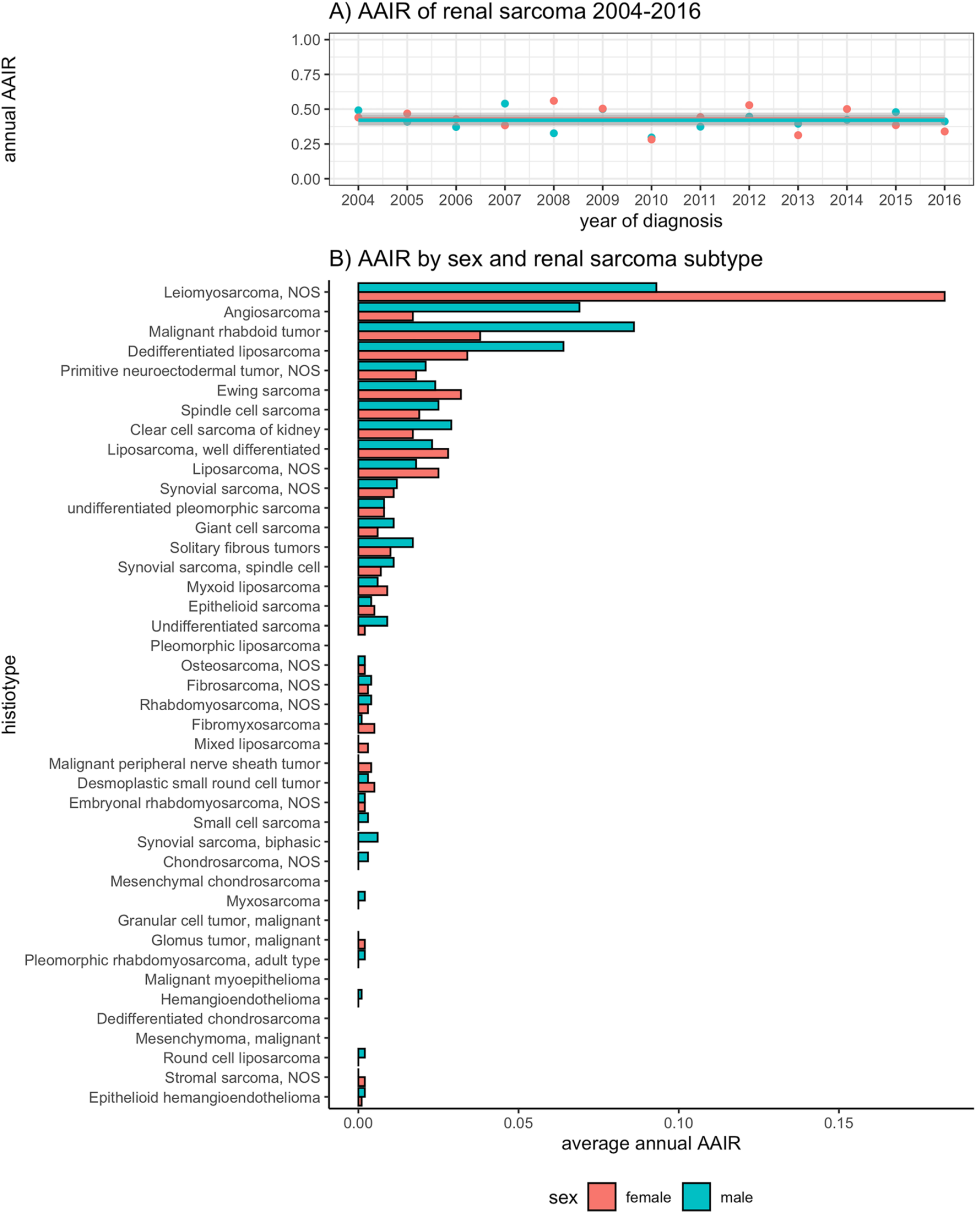
Age-adjusted incidence rates of renal sarcomas by sex, provided as cases/1 million. Empty rows indicate missing cases from the SEER database.

**Figure 2:**
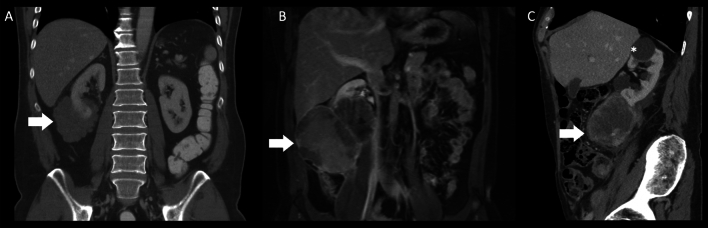
Representative radiological cases of primary renal angiosarcoma (**A**), primitive neuroectodermal tumor (PNET; **B**) and liposarcoma (**C**), each at the right lower renal pole. Subfigure A: coronal contrast-enhanced CT study demonstrating a lobulated, hypodense expophytic angiosarcoma (8.5 cm diameter; arrow). (**B**) coronal contrast-enhanced T1-weighted MR study showing an inhomogenously enhancing exophytic PNET (14.3 cm diameter; arrow). (**C**) sagittal contrast-enhanced CT study revealing a peripherally enhancing exophytic liposarcoma (8.7 cm diameter; arrow). A renal cyst is noted at the right upper renal pole (asterix).

**Figure 3:**
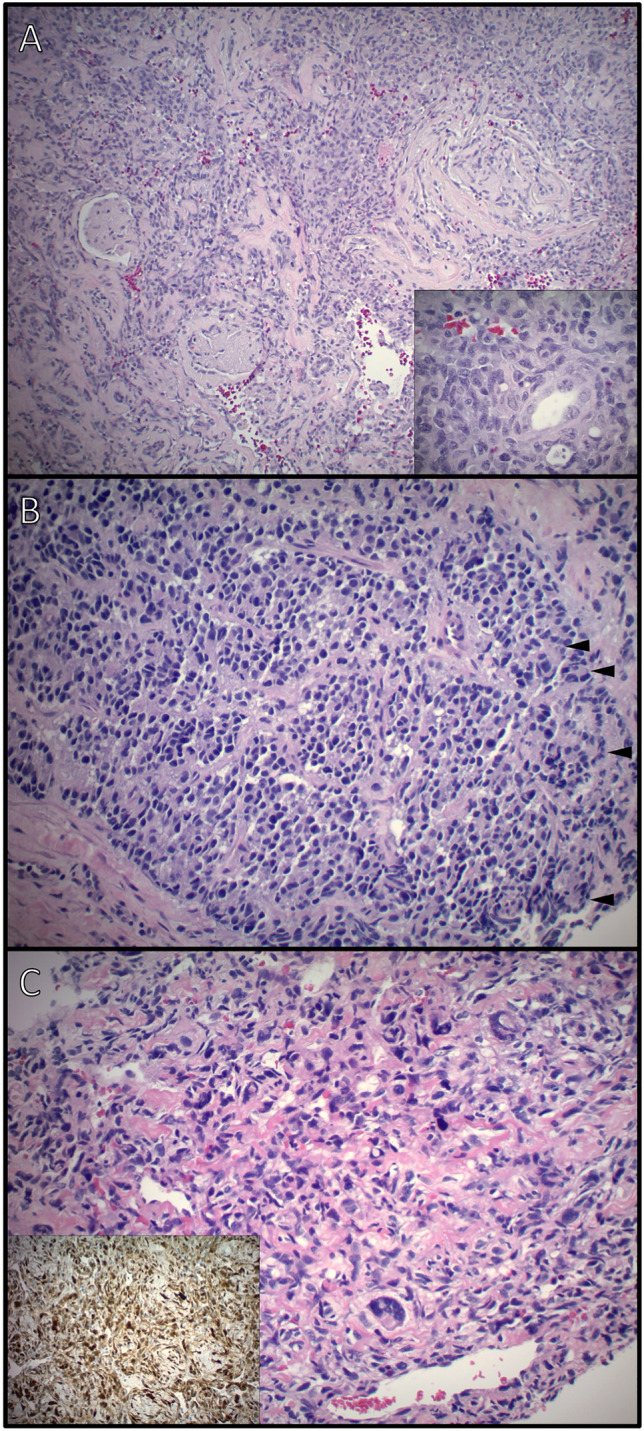
Representative histological cases of primary renal angiosarcoma (**A**), primitive neuroectodermal tumor (PNET; **B**) and dedifferentiated liposarcoma (**C**). (**A**) Section from a 19 cm renal angiosarcoma involving the renal cortex with entrapped glomeruli. Inset: Higher magnification (400×) demonstrating vascular spaces lined by atypical endothelial cells. (**B**) Core needle biopsy tissue demonstrating renal PNET with vaguely lobular growth of small round blue cells with hyperchromatic nuclei and pseudo-rossette formation (arrowheads). (**C**) Core needle biopsy tissue demonstrating dedifferentiated liposarcoma with pleomorphic spindle cells within a vascular and collagenous stroma. The tumor cells demonstrate cdk-4 (inset) and Rb expression by immunohistochemistry as well as mdm-2 amplification by fluorescence in situ hybridization.

The most frequent sarcoma histiotypes were leiomyosarcoma (LMS; 2004–2016 average annual AAIR: 0.14 cases/1 million) and malignant rhabdoid tumors (0.06 cases/1 million). Although no sex-specific difference in the overall AAIR was evident (male:female AAIR ratio = 1.04, *p* = 0.597; see Fig. 1), AAIR for sarcoma histiotypes varied by sex. For example, the male:female AAIR ratio was 0.46 for leiomyosarcoma (p < 0.001), but 3.82 for angiosarcoma (*p* < 0.001; see Fig. 1). Representative radiological and histological case studies are provided in Figs. 2 and 3.

Renal sarcoma incidence rates further varied according to age at diagnosis, with generally higher incidence rates for older patients (supplemental Fig. 2).

### Renal sarcoma presentation

The most frequent T stage of renal sarcomas was T3 (n = 426/1279; 31.9%), as detailed in Table 1. Synchronous distant metastases were reported in 32.2% of the patients (n = 412/1279). In 337 patients, the number and location of distant metastases was reported with pulmonary (n = 229/337; 68%) and osseous (n = 139/337; 41.2%) metastases being most common. Most of these patients presented with either 1 or 2 distant organ metastases (n = 171/337; 50.1%; and n = 98/337; 29.1%).

**Figure 4:**
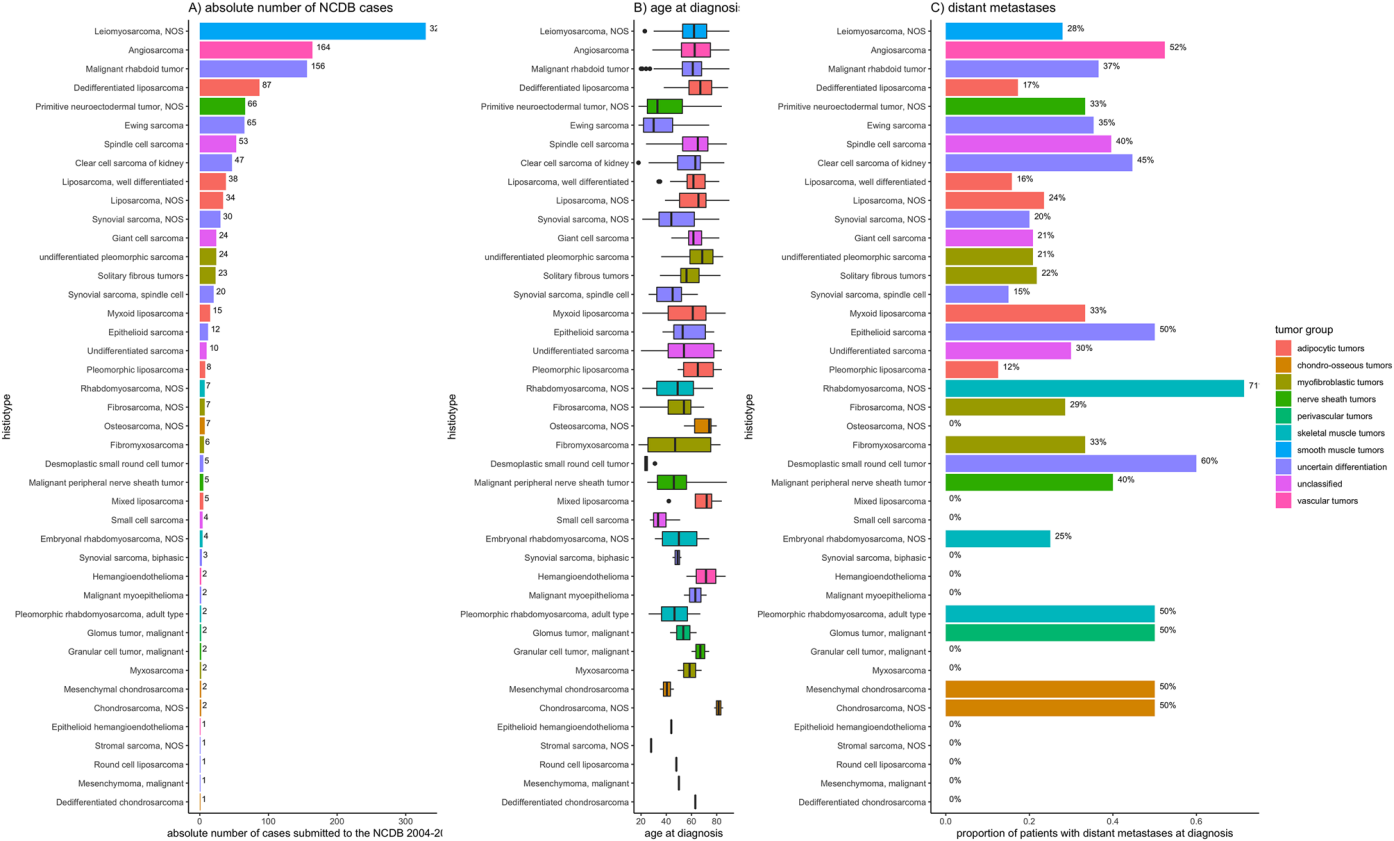
distribution of age and distant metastases at renal sarcoma diagnosis according to histiotype.

In the NCDB data, clinical presentation of renal sarcomas varied according to the 43 different histiotypes as depicted in Fig. 4 and supplemental Fig. 3. For example, median age at diagnosis of LMS was 62 years (IQR: 53–62 years) with median diameter of 86 mm (IQR: 50-141 mm), while median age at primitive neuroectodermal tumor (PNET) diagnosis was 33 years (IQR: 25–53 years) with median diameter of 100 mm (IQR: 80-142 mm). Further variation was evident regarding distant spread, e.g. with 28% of LMS cases (n = 92/239) presenting with distant metastases at time of diagnosis, compared to 52% of angiosarcoma cases (n = 86/164).

### Renal sarcoma treatment

While surgical resection was generally the cornerstone of renal sarcoma therapy, treatment variability was seen according to tumor stage and metastatic status, as summarized in Table 2 and supplemental Table 2.

**Table 2:** Treatment variables of included NCDB patients.

Parameter	Level	Total	No metastases reported	Distant metastases	*p* value
n		1279	867	412	
Renal surgery					< *0.01*
	Total nephrectomy	886 (69.3%)	670 (77.3%)	216 (52.4%)	
	Extended nephrectomy	65 (5.1%)	42 (4.8%)	23 (5.6%)	
	Partial nephrectomy	74 (5.8%)	61 (7.0%)	13 (3.2%)	
	Local tumor destruction	8 (0.6%)	7 (0.8%)	1 (0.2%)	
	Surgery, NOS	11 (0.9%)	6 (0.7%)	5 (1.2%)	
	No surgery	235 (18.4%)	81 (9.3%)	154 (37.4%)	
Regional lymph node surgery					0.37
	No regional lymph node surgery	901 (70.4%)	600 (69.2%)	301 (73.1%)	
	Regional lymph node surgery	357 (27.9%)	252 (29.1%)	105 (25.5%)	
	Unknown if there was any Regional lymph node surgery	21 (1.6%)	15 (1.7%)	6 (1.5%)	
Resection margin					< *0.01*
	R0	727 (69.6%)	573 (72.9%)	154 (59.7%)	
	R +	222 (21.3%)	139 (17.7%)	83 (32.2%)	
	RX	95 (9.1%)	74 (9.4%)	21 (8.1%)	
Systemic therapy					< *0.01*
	No systemic therapy	868 (67.9%)	681 (78.5%)	187 (45.4%)	
	Systemic therapy	411 (32.1%)	186 (21.5%)	225 (54.6%)	
Radiation					< *0.01*
	Primary site radiation	66 (5.2%)	57 (6.6%)	9 (2.2%)	
	metastatic site radiation	118 (9.2%)	0 (0.0%)	118 (28.6%)	
	No radiation	1095 (85.6%)	810 (93.4%)	285 (69.2%)	

The majority of renal sarcomas were treated with radical nephrectomy (n = 886/1279; 69.3%). Among T4 renal sarcomas (n = 181/1279; 14.2%), radical nephrectomy and extended nephrectomy were performed in 53.6% (n = 97/181) and 13.8% (n = 25/181), respectively. In metastatic cases (n = 412), radical nephrectomy was used in 52.4% (n = 216/412). Among patients with non-pulmonary sarcoma metastases, any surgical resection was done in 58.3% of cases (n = 74/127). Regional lymph node resection was performed in 33.9% of all surgically treated patients (n = 354/1044).

While surgical margins were assessed as R + in 21.3% among all surgically treated patients (n = 222/1044), a higher frequency of positive surgical margins was reported in T4 sarcoma cases undergoing surgical treatment (n = 52/128; 40.6%).

Systemic therapy was administered in 16.1% of the patients with localized renal sarcoma (n = 65/403 among T1/2 sarcomas without metastases). A higher proportion of systemic therapy was used in patients with locally advanced sarcomas (n = 72/181 T4 sarcomas; 39.8%) and distant metastases (n = 225/412; 54.6%), those with positive surgical margins (n = 67/222; 30.2%) and among cases with tumor necrosis (n = 63/222; 34.6%).

For 282 patients, data on sequencing of surgical treatment and systemic therapy was available: in the majority of these patients, systemic therapy was used in an adjuvant setting (n = 263/282; 93.3%), and less commonly in a neoadjuvant (n = 13/282; 4.6%).

Only a minority of patients received radiation of renal sarcoma primary site (n = 66/1279; 5.2%) or synchronous metastases (n = 118/1279; 9.2%).

### Renal sarcoma outcome

Data on survival time and survival status was available for n = 1177 NCDB renal sarcoma patients. In this cohort, median follow-up time was 77.7 months (IQR: 39.8–109 months).

The median OS for all renal sarcomas was 25 months, but OS rates varied according to sarcoma histiotype, as demonstrated in supplemental Table 3.

**Figure 5:**
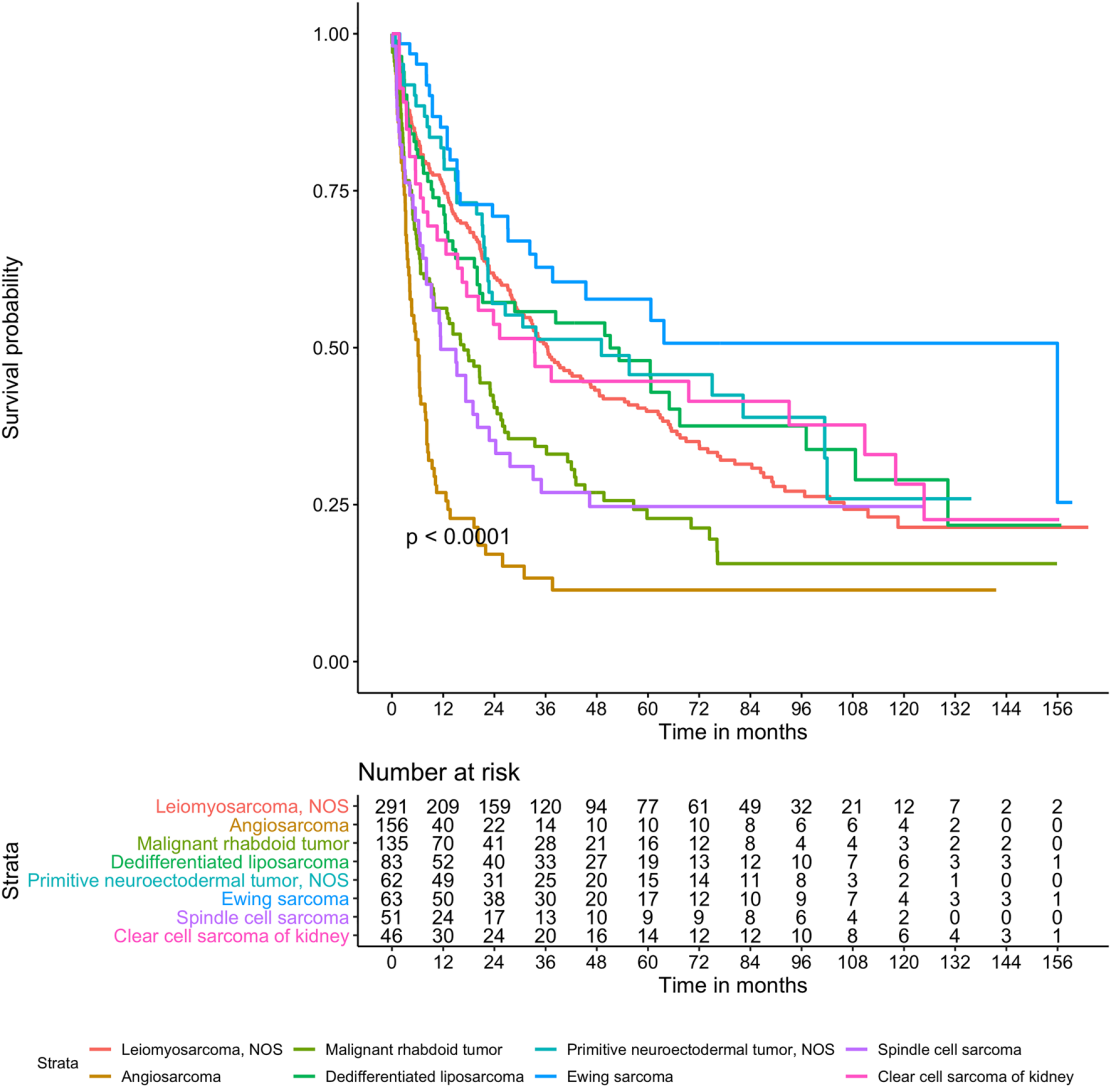
differences in overall survival according to renal sarcoma histiotype.

As detailed in Fig. 5 and supplemental Table 4, OS independently varied with sarcoma histiotype on multivariable analyses: shorter OS was observed in patients with angiosarcomas compared to those with LMS (HR = 2.42, 95% CI 1.89–3.1, *p* < 0.001).

In multivariable analyses, longer OS was reported in younger female patients with lower T stage, without histological tumor necrosis and distant metastases, as well as patients receiving surgical treatment and systemic therapy (multivariable *p* < 0.05, each).

Due to statistical collinearity with surgical treatment, the OS effect of surgical margin was separately assessed in surgically resected patients, where renal sarcomas with positive surgical margin independently demonstrated shorter OS versus R0 resected sarcomas (R + vs. R0, HR = 1.36, 95% CI 1.1–1.68, *p* = 0.004; see supplemental Table 5).

### Sarcoma histiotype-specific treatment outcome

On multivariable OS models, a statistically significant interaction between sarcoma treatment and histiotype was evident, indicating varying effectiveness of systemic therapy (interaction *p* < 0.001) and surgical resection (interaction *p* < 0.001) according to sarcoma histiotype.

Supplemental Fig. 4 details this heterogeneity, demonstrating longer OS for systemic therapy versus none in patients with LMS, angiosarcoma and clear cell sarcoma (*p* < 0.05, respectively) after multivariable adjustment.

Longer OS for surgical resection versus none was observed in LMS, malignant rhabdoid tumors, PNET, Ewing sarcoma, and spindle cell sarcoma (*p* < 0.05, respectively).

## Discussion

Sarcomas are a heterogenous group of tumors that manifest throughout the body, varying in their clinical presentation, behavior and outcome^5^.

Using the SEER and NCDB databases, we demonstrated that renal sarcomas comprise 0.25% of all renal malignancies in the US with peaking incidence in patients age 80 years and without relevant changes in incidence from 2004 to 2016. A total of 43 different renal sarcoma histiotypes were reported, the most common being LMS and malignant rhabdoid tumors. Renal sarcomas manifested at a median age of 60 years, with a median diameter of 10 cm, and with metastases in 29.1% of cases, of which the majority were pulmonary. Still, considerable variability of demographic parameters and clinical presentation was evident according to the sarcomas histiotype.

Renal sarcomas seem to resemble retroperitoneal sarcomas from an epidemiological point of view: data on retroperitoneal STS diagnosed from 1973 to 2001 reported no temporal changes in incidence^15^, while analyses from the French network of cancer registries support a female LMS predominance^16^. Further, the heterogeneity of sarcoma histiotypes with varying clinical presentation is well described in the current literature, although most studies tend to focus on STS of the extremities and trunk without specific analyses for renal sarcomas^5,6,17^.

This analysis found that surgical resection was the cornerstone of renal sarcoma treatment, with almost 70% of NCDB cases receiving radical nephrectomy. Systemic therapy alone or in combination was mainly used for locally advanced renal sarcomas or patients with distant metastases, while radiation for the primary site was performed only in a minority of patients (5.2%). Although surgical resection and systemic therapy were generally associated with longer OS on multivariable analyses, their effectiveness varied according to sarcoma histiotype. These results indicate a variable sensitivity to systemic therapy according to histiotype, highlighting the importance of histiotype-specific treatment approachs for renal sarcoma, which is in line with previous observations in STS^18,19^.

Real-world renal sarcoma treatment patterns seem to follow guideline recommendations for renal cell carcinoma, advocating surgical resection with curative intent for early-stage renal cancer, and systemic therapy for metastatic disease, which can be accompanied by cytoreductive surgery in selected patients^20,21^. Although recent trials have questioned the role of cytoreductive nephrectomy in the setting of systemic therapy advances^22,23^, the NCDB data reported up to 2016 most likely does not reflect these developments. In contrast to renal cancer guidelines, the ESMO guideline on visceral and soft tissue sarcomas advocate risk-adapted perioperative treatments in sensitive histotypes, which may involve perioperative chemotherapy and/or radiation^24^. Non-superficial, large sarcoma size (> 5 cm) and grade 2 or 3 are considered key factors to define high-risk, which render patients candidates for multimodal therapy^24^.

However, among comparable renal sarcoma patients, less than 55% received systemic therapy and less than 6% radiation of the primary site, which may indicate an underutilization of multimodal therapy in these patients.

Although resection of pulmonary metastases with or without prior chemotherapy is considered standard of care according to ESMO guidelines^24^, the sole use of systemic therapy is recommended in cases with extra-pulmonary metastases. In the NCDB, the majority of renal sarcoma patients with extra-pulmonary metastases were treated with surgical resection with or without systemic therapy (58.3%).

Both of these findings corroborate that real-world renal sarcoma treatment resembles that of renal cancer and not that of STS. To individualize and optimize multidisciplinary treatment planning, pre-treatment tumor biopsies may play a clinical role for renal sarcoma patients, which would follow guidelines recommending tumor biopsies for the management of retroperitoneal STS. While renal tumors with classical radiological findings often undergo upfront surgical resection, biopsies might be benefitial in patients presenting with atypical renal tumors. For example, patients with very large, heterogeneous renal tumors and infiltration of adjacent organs have a higher probability of renal sarcoma, as reported in an earlier study, and might thus benefit from a tumor biopsy^25^.

The distinction of renal sarcoma and retroperitoneal STS is a diagnostic challenge, both for radiologists and pathologsits. While some imaging markers might suggest a renal origin, such as the so-called claw-sign on cross-sectional radiological imaging^26^, large tumor diameter and perifocal tumor infiltration might complicate the diagnosis. The comparable epidemiology of renal sarcomas and retroperitoneal STS observed in this study might be further evidence that misclassifications could have occurred in the NCDB and SEER databases. In particular, the retrospective character of theses databases did not allow for a case review by reference pathologists or experienced GU-radiologists.

While this study revealed a median overall survival of 25 months for renal sarcomas, OS rates showed relevant variability according to sarcoma histiotype, as well as treatment approach (multivariable *p* < 0.05). Patients who received multimodal therapy, consisting of surgery and chemotherapy, achieved significant better OS, suggesting that STS guidelines might be appropriate for renal sarcoma treatment planning.

Although sarcoma grade was not available from the NCDB in the majority of cases, histological tumor necrosis was used as a surrogate parameter and emergerd as a negative independent OS predictor for renal sarcomas.

Several of the OS predictors for renal sarcomas identified in this study have been described for STS as well. For example, sarcoma histiotype, patient age, tumor extent, FNCLCC grading, as well as surgical resection margins are components of nomograms for prediction of overall survival and disease-specific death in STS patients^27–29^.

This study is not devoid of limitations. First, despite information on the histiotype, no details were available on the mutational status of renal sarcomas. Second, histological grading was not reported for the majority of renal sarcomas in the NCDB, although the presence of tumor necrosis may serve as a surrogate parameter. Third, the inaccuracy of histopathology in the absence of central review is well-recognized^18^.

Furthermore, no data on cancer-specific and recurrence-free survival were available from the NCDB. Since only a minority of renal sarcoma patients received radiation therapy, no conclusive analyses regarding the effectiveness of local radiation were possible in this study. Given the similarities between renal sarcomas and retroperitoneal STS observed in this study, further trials are warranted to evaluate radiation therapy protocols for patients presenting with renal sarcomas. Finally, details on systemic therapy regimens were unavailable from the NCDB, which prevented analyses of the clinical effectiveness of specific systemic therapy protocols.

## Conclusions

This analysis demonstrates that renal sarcomas comprise 0.25% of all renal malignancies in the US with constant incidence rates from 2004 to 2016. Renal sarcoma histiotypes show considerable heterogeneity regarding epidemiology, clinical presentation, outcome and sensitivity to systemic therapy, and thus should be considered separate entities with individualized treatment approaches.

Although similar to retroperitoneal STS, renal sarcoma real-world treatment patterns closely resemble those recommended by renal cancer guidelines. In turn, this may indicate suboptimal utilization of multimodal therapy in selected renal sarcoma patients, which is considered the standard-of-care by visceral and soft tissue sarcoma guidelines. Given the variability of renal sarcoma therapy options and efficacy, a multidisciplinary treatment approach at specialized centers should be considered to optimize patient outcomes.

Future studies are needed to assess the efficacy of specific systemic therapy protocols in renal sarcomas, as well as to evaluate local and distant control rates.

### Supplementary Information

Supplementary Information.

## Data Availability

The data assessed in this study are available from the NCDB and the SEER database upon request.

## References

[1] Bray F (2018). Global cancer statistics 2018: GLOBOCAN estimates of incidence and mortality worldwide for 36 cancers in 185 countries. CA Cancer J. Clin..

[2] Hollingsworth JM (2006). Rising incidence of small renal masses: a need to reassess treatment effect. J. Natl. Cancer Inst..

[3] Kane CJ (2008). Renal cell cancer stage migration: Analysis of the National Cancer Data Base. Cancer.

[4] Nguyen, M.M., Gill, I.S., & Ellison, L.M. The evolving presentation of renal carcinoma in the United States: Trends from the Surveillance, Epidemiology, and End Results program*.* J Urol, **176**(6 Pt 1), pp. 2397–400 (2006); discussion 2400. 10.1016/j.juro.2006.07.144 17085111

[5] Gamboa AC, Gronchi A, Cardona K (2020). Soft-tissue sarcoma in adults: An update on the current state of histiotype-specific management in an era of personalized medicine. CA Cancer J. Clin..

[6] Fletcher, C.D.M., World Health Organization., and International Agency for Research on Cancer., *WHO classification of tumours of soft tissue and bone*. 4th ed. World Health Organization classification of tumours. 2013, Lyon: IARC Press. 468 p.

[7] Wang X (2011). Adult renal sarcoma: Clinical features and survival in a series of patients treated at a high-volume institution. Urology.

[8] Öztürk H (2015). Prognostic features of renal sarcomas (Review). Oncol. Lett..

[9] Katabathina VS (2010). Mesenchymal neoplasms of the kidney in adults: Imaging spectrum with radiologic-pathologic correlation. RadioGraphics.

[10] Lalwani N (2011). Pediatric and adult primary sarcomas of the kidney: A cross-sectional imaging review. Acta Radiol..

[11] Karaosmanoğlu AD (2015). Unusual malignant solid neoplasms of the kidney: Cross-sectional imaging findings. Korean J. Radiol..

[12] Fremgen AM (1999). Clinical highlights from the National Cancer Data Base, 1999. CA Cancer J. Clin..

[13] Surveillance, E., & End Results (SEER) Program (www.seer.cancer.gov) SEER*Stat Database. *Incidence—SEER Research Limited-Field Data, 21 Registries, Nov 2019 Sub (2000–2017) —Linked To County Attributes—Time Dependent (1990–2017) Income/Rurality, 1969–2017 Counties, National Cancer Institute, DCCPS, Surveillance Research Program, released April 2020, based on the November 2019 submission.* 2020 10/15/2020].

[14] Schemper M (1992). Cox analysis of survival data with non-proportional hazard functions. J. R. Stat. Soc..

[15] Porter GA, Baxter NN, Pisters PW (2006). Retroperitoneal sarcoma: A population-based analysis of epidemiology, surgery, and radiotherapy. Cancer.

[16] Amadeo B (2020). Incidence and time trends of sarcoma (2000–2013): Results from the French network of cancer registries (FRANCIM). BMC Cancer.

[17] Trama A (2019). Soft tissue sarcoma in Italy: From epidemiological data to clinical networking to improve patient care and outcomes. Cancer Epidemiol..

[18] Young RJ (2017). Predictive and prognostic factors associated with soft tissue sarcoma response to chemotherapy: A subgroup analysis of the European Organisation for Research and Treatment of Cancer 62012 study. Acta Oncol..

[19] Glabbeke MV (1999). Prognostic factors for the outcome of chemotherapy in advanced soft tissue sarcoma: An analysis of 2185 patients treated with anthracycline-containing first-line regimens—A European organization for research and treatment of cancer soft tissue and bone sarcoma group study. J. Clin. Oncol..

[20] Ljungberg B (2019). European association of urology guidelines on renal cell carcinoma: The 2019 update. Eur. Urol..

[21] Campbell S (2017). Renal mass and localized renal cancer: AUA guideline. J Urol.

[22] Psutka SP (2019). Reassessing the role of cytoreductive nephrectomy for metastatic renal cell carcinoma in 2019. Am. Soc. Clin. Oncol. Educ. Book.

[23] Méjean A (2018). sunitinib alone or after nephrectomy in metastatic renal-cell carcinoma. N. Engl. J. Med..

[24] Casali PG (2018). Soft tissue and visceral sarcomas: ESMO-EURACAN Clinical Practice Guidelines for diagnosis, treatment and follow-up. Ann. Oncol..

[25] Uhlig J (2022). Primary renal sarcomas: Imaging features and discrimination from non-sarcoma renal tumors. Eur. Radiol..

[26] Combrink L, Beviss-Challinor KB (2021). Magnetic resonance imaging for paediatric retroperitoneal masses: Diagnostic accuracy of the claw sign. SA J. Radiol..

[27] Ardoino I (2010). Histology-specific nomogram for primary retroperitoneal soft tissue sarcoma. Cancer.

[28] Gronchi A (2013). Outcome prediction in primary resected retroperitoneal soft tissue sarcoma: Histology-specific overall survival and disease-free survival nomograms built on major sarcoma center data sets. J. Clin. Oncol..

[29] Tan MC (2016). Histology-based classification predicts pattern of recurrence and improves risk stratification in primary retroperitoneal sarcoma. Ann Surg.

