# Author Correction: Epidemiology, treatment and outcomes of primary renal sarcomas in adult patients

**DOI:** 10.1038/s41598-024-62573-3

**Published:** 2024-05-24

**Authors:** Johannes Uhlig, Annemarie Uhlig, Hari Deshpande, Philipp Ströbel, Lutz Trojan, Joachim Lotz, Michael Hurwitz, Omeed Hafez, Peter Humphrey, Viktor Grünwald, Hyun S. Kim

**Affiliations:** 1 Department of Diagnostic and Interventional Radiology, University Medical Center Goettingen, Robert-Koch-Strasse 40, 37075 Göttingen, Germany; 2 Department of Diagnostic Radiology and Nuclear Imaging, University of Maryland School of Medicine, Baltimore, MD USA; 3 Department of Urology, University Medical Center Goettingen, Göttingen, Germany; 4 Institute of Urologic Oncology, University of California at Los Angeles, Los Angeles, CA USA; 5 Smilow Cancer Hospital, New Haven, CT USA; 6 Department of Pathology, University Medical Center Goettingen, Göttingen, Germany; 7 Department of Pathology, Yale School of Medicine, New Haven, CT USA; 8 Clinic for Medical Oncology and Clinic for Urology, University Hospital Essen, Essen, Germany

Correction to: *Scientific Reports* 10.1038/s41598-024-60174-8, published online 02 May 2024

The Acknowledgements section in the original version of this Article was omitted. It now reads:

Acknowledgements

“We acknowledge support by the Open Access Publication Funds/transformative agreements of the Göttingen University.”

The original Article has been corrected.

