# Supplementary material for: Epidemiology, treatment and outcomes of primary renal sarcomas in adult patients
Source: Sci Rep. 2024 May 2;14:10038. doi: 10.1038/s41598-024-60174-8 (PMC11063157; doi:10.1038/s41598-024-60174-8)
Supplement: Supplementary file 1 — Supplementary Information. [file 41598_2024_60174_MOESM1_ESM.docx]

**Supplemental material**

Methodological details on NCDB variables

NCDB data were collected at the patient level (age, sex, race, comorbidities measured by Charlson-Deyo Comorbidity index), hospital-level (academic vs non-academic, facility location), and tumor-level (year of diagnosis, tumor diameter, T stage, presence and location of metastases at time of diagnosis, histological tumor necrosis, surgical treatment, surgical margins, systemic therapy). The NCDB did not disclose facility type and location for patients age <39 years to ensure anonymization in this defined cohort. Cancer tumor stage (T stage) was determined according to kidney cancer staging in the 8^th^ edition of the UICC, summarized as T1, T2, T3, T4 and TX. The T stage is a composite parameter comprising tumor diameter as well as local tumor infiltration.

Since histopathological data on N status were available for less than 7% of renal sarcoma patients from the NCDB, N status was not assessed in this study. Cancer diameter was measured in millimeter at time of diagnosis. Renal sarcoma metastases were reported as presence or absence of distant organ metastases at time of diagnosis. Metastatic location was provided as osseous, hepatic, cerebral, or pulmonary. No information was provided on distant adrenal gland metastases. In cases with radiation to distant sites (i.e. brain), distant metastases were assumed even if not coded by the NCDB.

Given that renal sarcoma histology grade according to ICD-03 was only reported in 37% of NCDB patients and no sufficient information was provided to calculate internationally recognized systems such as the French Federation of Cancer Centers Sarcoma Group (FNCLCC) grade, tumor grade was not evaluated in this study. Histologically assessed renal sarcoma necrosis was stratified as “necrosis” and “no necrosis”, conservatively including cases with unknown status as “no necrosis”.

Overall survival was defined as time from renal sarcoma diagnosis to death or censoring and calculated from the NCDB as this database provided larger sample sizes compared to the SEER cohort, facilitating analyses in rare renal sarcoma subgroups.

Given recent changes in WHO coding for sarcomas, “malignant fibrous histiocytomas” were recoded as “undifferentiated pleomorphic sarcomas”, and “hemangioenotheliomas” as “angiosarcomas”.

Since the NCDB does not distinguish between different systemic treatment approaches, such as cytotoxic chemotherapy or targeted therapy, the general term “systemic therapy” was used.

Radiotherapy was stratified as “primary site radiation” (including patients receiving radiotherapy with primary volume “Kidney” or “Abdomen, NOS”), “metastatic site radiation”, and “no radiation”.

Surgical margins were classified as R0, R+ (combined R1 or R2), and RX. Lymph node resection was reported as “regional lymph node resection”.

Supplemental table 1: cellular origin of sarcoma histiotypes.

| **celluar origin** | **sarcoma histiotype** | **ICD-O3 code** |
| --- | --- | --- |
| adipocytic tumors | Liposarcoma, NOS | 8850 |
| adipocytic tumors | Liposarcoma, well differentiated | 8851 |
| adipocytic tumors | Myxoid liposarcoma | 8852 |
| adipocytic tumors | Pleomorphic liposarcoma | 8854 |
| adipocytic tumors | Mixed liposarcoma | 8855 |
| adipocytic tumors | Dedifferentiated liposarcoma | 8858 |
| chondro-osseous tumors | Osteosarcoma, NOS | 9180 |
| chondro-osseous tumors | Chondrosarcoma, NOS | 9220 |
| chondro-osseous tumors | Mesenchymal chondrosarcoma | 9240 |
| chondro-osseous tumors | Dedifferentiated chondrosarcoma | 9243 |
| myofibroblastic tumors | Fibrosarcoma, NOS | 8810 |
| myofibroblastic tumors | Fibromyxosarcoma | 8811 |
| myofibroblastic tumors | Solitary fibrous tumors | 8815 |
| myofibroblastic tumors | undifferentiated pleomorphic sarcoma | 8830 |
| myofibroblastic tumors | Myxosarcoma | 8840 |
| myofibroblastic tumors | Solitary fibrous tumors | 9150 |
| nerve sheath tumors | Primitive neuroectodermal tumor, NOS | 9473 |
| nerve sheath tumors | Malignant peripheral nerve sheath tumor | 9540 |
| nerve sheath tumors | Granular cell tumor, malignant | 9580 |
| perivascular tumors | Glomus tumor, malignant | 8711 |
| skeletal muscle tumors | Rhabdomyosarcoma, NOS | 8900 |
| skeletal muscle tumors | Pleomorphic rhabdomyosarcoma, adult type | 8901 |
| skeletal muscle tumors | Embryonal rhabdomyosarcoma, NOS | 8910 |
| smooth muscle tumors | Leiomyosarcoma, NOS | 8890 |
| uncertain differentiation | Epithelioid sarcoma | 8804 |
| uncertain differentiation | Desmoplastic small round cell tumor | 8806 |
| uncertain differentiation | Round cell liposarcoma | 8853 |
| uncertain differentiation | Stromal sarcoma, NOS | 8935 |
| uncertain differentiation | Malignant rhabdoid tumor | 8963 |
| uncertain differentiation | Clear cell sarcoma of kidney | 8964 |
| uncertain differentiation | Malignant myoepithelioma | 8982 |
| uncertain differentiation | Mesenchymoma, malignant | 8990 |
| uncertain differentiation | Synovial sarcoma, NOS | 9040 |
| uncertain differentiation | Synovial sarcoma, spindle cell | 9041 |
| uncertain differentiation | Synovial sarcoma, biphasic | 9043 |
| uncertain differentiation | Ewing sarcoma | 9260 |
| unclassified | Spindle cell sarcoma | 8801 |
| unclassified | Giant cell sarcoma | 8802 |
| unclassified | Small cell sarcoma | 8803 |
| unclassified | Undifferentiated sarcoma | 8805 |
| vascular tumors | Angiosarcoma | 9120 |
| vascular tumors | Hemangioendothelioma | 9130 |
| vascular tumors | Epithelioid hemangioendothelioma | 9133 |

Supplemental table 2: primary treatment strategies according to renal sarcoma T stage and distant metastases in the NCDB. Patients with TX sarcomas were excluded for these analyses.

| treatment | total | T1-2, no distant metastases | T1-2, distant metastases | T3/4, no distant metastases | T3/4, distant metastases |
| --- | --- | --- | --- | --- | --- |
| total n | 1136 | 422 | 107 | 394 | 213 |
| surgery + systemic therapy | 267 (23.5%) | 62 (14.7%) | 30 (28.0%) | 92 (23.4%) | 83 (39.0%) |
| surgery alone | 682 (60.0%) | 319 (75.6%) | 18 (16.8%) | 274 (69.5%) | 71 (33.3%) |
| systemic therapy alone | 94 (8.3%) | 10 (2.4%) | 33 (30.8%) | 14 (3.6%) | 37 (17.4%) |
| no surgery or systemic therapy | 93 (8.2%) | 31 (7.3%) | 26 (24.3%) | 14 (3.6%) | 22 (10.3%) |

Supplemental table 3: detailed overall survival (OS) rates and 95% confidence intervals estimated from Kaplan-Meier curves. Two patients with mesenchymal chondrosarcoma (ICD-03 code 9240) were not included since no OS time was reported.

| ICD-03 code | histiotype | tumor group | number of NCDB cases | 1-year OS rate | 2-year OS rate | 3-year OS rate | 4-year OS rate | 5-year OS rate |
| --- | --- | --- | --- | --- | --- | --- | --- | --- |
| 8890 | Leiomyosarcoma, NOS | smooth muscle tumors | 329 | 75.7% | 61.5% | 50.6% | 43.2% | 39.9% |
| 9120 | Angiosarcoma | vascular tumors | 164 | 26.9% | 17.1% | 13.3% | 11.4% | 11.4% |
| 8963 | Malignant rhabdoid tumor | uncertain differentiation | 156 | 56.3% | 40.5% | 34.3% | 26.9% | 22.8% |
| 8858 | Dedifferentiated liposarcoma | adipocytic tumors | 87 | 72.6% | 57.2% | 55.7% | 53.9% | 47.9% |
| 9473 | Primitive neuroectodermal tumor, NOS | nerve sheath tumors | 66 | 81.8% | 57% | 51.3% | 51.3% | 45.7% |
| 9260 | Ewing sarcoma | uncertain differentiation | 65 | 85.1% | 70.9% | 62.8% | 57.7% | 57.7% |
| 8801 | Spindle cell sarcoma | unclassified | 53 | 49.7% | 35.2% | 26.9% | 24.7% | 24.7% |
| 8964 | Clear cell sarcoma of kidney | uncertain differentiation | 47 | 67.1% | 53.7% | 47% | 44.6% | 44.6% |
| 8851 | Liposarcoma, well differentiated | adipocytic tumors | 38 | 88.2% | 75% | 67.3% | 43.3% | 43.3% |
| 8850 | Liposarcoma, NOS | adipocytic tumors | 34 | 79.3% | 68.4% | 60.8% | 60.8% | 60.8% |
| 9040 | Synovial sarcoma, NOS | uncertain differentiation | 30 | 69% | 48.3% | 34.1% | 30.3% | 17.3% |
| 8802 | Giant cell sarcoma | unclassified | 24 | 68.2% | 39.2% | 32.6% | 26.1% | 17.4% |
| 8830 | undifferentiated pleomorphic sarcoma | myofibroblastic tumors | 24 | 61.7% | 51.4% | 51.4% | 46.3% | 46.3% |
| 9041 | Synovial sarcoma, spindle cell | uncertain differentiation | 20 | 100% | 57.9% | 42.1% | 35.1% | 26.3% |
| 8815 | Solitary fibrous tumors | myofibroblastic tumors | 15 | 81% | 76.2% | 76.2% | 71.1% | 65.2% |
| 8852 | Myxoid liposarcoma | adipocytic tumors | 15 | 73.3% | 73.3% | 53.3% | 53.3% | 44.4% |
| 8804 | Epithelioid sarcoma | uncertain differentiation | 12 | 60% | 60% | 40% | 40% | 40% |
| 8805 | Undifferentiated sarcoma | unclassified | 10 | 40% | 30% | 30% | 30% | 30% |
| 8854 | Pleomorphic liposarcoma | adipocytic tumors | 8 | 87.5% | 72.9% | 72.9% | 43.8% | 43.8% |
| 9150 | Solitary fibrous tumors | myofibroblastic tumors | 8 | 81% | 76.2% | 76.2% | 71.1% | 65.2% |
| 8810 | Fibrosarcoma, NOS | myofibroblastic tumors | 7 | 51.4% | 34.3% | 17.1% | 17.1% | 17.1% |
| 8900 | Rhabdomyosarcoma, NOS | skeletal muscle tumors | 7 | 60% | 60% | 20% | 20% | 20% |
| 9180 | Osteosarcoma, NOS | chondro-osseous tumors | 7 | 60% | 60% | 60% | 60% | 60% |
| 8811 | Fibromyxosarcoma | myofibroblastic tumors | 6 | 83.3% | 83.3% | 83.3% | 83.3% | 83.3% |
| 8806 | Desmoplastic small round cell tumor | uncertain differentiation | 5 | 50% | 50% | 50% | NA | NA |
| 8855 | Mixed liposarcoma | adipocytic tumors | 5 | 60% | 60% | 40% | 20% | 20% |
| 9540 | Malignant peripheral nerve sheath tumor | nerve sheath tumors | 5 | 40% | 40% | NA | NA | NA |
| 8803 | Small cell sarcoma | unclassified | 4 | 75% | 75% | NA | NA | NA |
| 8910 | Embryonal rhabdomyosarcoma, NOS | skeletal muscle tumors | 4 | 25% | 25% | 25% | 25% | 25% |
| 9043 | Synovial sarcoma, biphasic | uncertain differentiation | 3 | 100% | 100% | 50% | 50% | 50% |
| 8711 | Glomus tumor, malignant | perivascular tumors | 2 | NA | NA | NA | NA | NA |
| 8840 | Myxosarcoma | myofibroblastic tumors | 2 | 100% | 100% | 100% | 100% | 100% |
| 8901 | Pleomorphic rhabdomyosarcoma, adult type | skeletal muscle tumors | 2 | 100% | 50% | 50% | 50% | 50% |
| 8982 | Malignant myoepithelioma | uncertain differentiation | 2 | NA | NA | NA | NA | NA |
| 9130 | Hemangioendothelioma | vascular tumors | 2 | 50% | 50% | 50% | 50% | 50% |
| 9220 | Chondrosarcoma, NOS | chondro-osseous tumors | 2 | NA | NA | NA | NA | NA |
| 9240 | Mesenchymal chondrosarcoma | chondro-osseous tumors | 2 | NA | NA | NA | NA | NA |
| 9580 | Granular cell tumor, malignant | nerve sheath tumors | 2 | 100% | 100% | 100% | 100% | 100% |
| 8853 | Round cell liposarcoma | uncertain differentiation | 1 | NA | NA | NA | NA | NA |
| 8935 | Stromal sarcoma, NOS | uncertain differentiation | 1 | 100% | 100% | 100% | 100% | 100% |
| 8990 | Mesenchymoma, malignant | uncertain differentiation | 1 | 100% | NA | NA | NA | NA |
| 9133 | Epithelioid hemangioendothelioma | vascular tumors | 1 | 100% | 100% | NA | NA | NA |

Supplemental table 4: univariate and multivariable weighted Cox proportional hazards models predicting overall survival. * Surgical margin was not included in multivariable analyses due to statistical collinearity with surgical resection.

| variable | level | univariate HR | multivariable HR |
| --- | --- | --- | --- |
| age | continuous | 1.01 (1.01-1.02), p<0.001 | 1.01 (1.01-1.02), p<0.001 |
| gender | male | 1 (reference) | 1 (reference) |
|  | female | 0.64 (0.55-0.74), p<0.001 | 0.83 (0.7-0.98), p=0.03 |
| race | Caucasian | 1 (reference) | - |
|  | African American | 0.73 (0.55-0.96), p=0.025 | - |
|  | others | 0.72 (0.5-1.04), p=0.079 | - |
| comorbidities (Charlson Deyo Comorbidity Index) | 0 | 1 (reference) | 1 (reference) |
|  | >=1 | 1.31 (1.11-1.54), p=0.002 | 1.28 (1.07-1.53), p=0.007 |
| renal sarcoma histiotype | Leiomyosarcoma, NOS | 1 (reference) | 1 (reference) |
|  | Angiosarcoma | 3.15 (2.42-4.1), p<0.001 | 2.42 (1.89-3.1), p<0.001 |
|  | Malignant rhabdoid tumor | 1.66 (1.27-2.17), p<0.001 | 1.31 (0.97-1.76), p=0.079 |
|  | Dedifferentiated liposarcoma | 0.95 (0.67-1.35), p=0.775 | 0.93 (0.62-1.39), p=0.721 |
|  | Primitive neuroectodermal tumor, NOS | 0.86 (0.6-1.23), p=0.411 | 1.23 (0.83-1.81), p=0.307 |
|  | Ewing sarcoma | 0.77 (0.45-1.33), p=0.348 | 1.26 (0.74-2.16), p=0.396 |
|  | Spindle cell sarcoma | 1.67 (1.11-2.51), p=0.014 | 1.41 (0.91-2.17), p=0.12 |
|  | Clear cell sarcoma of kidney | 1.05 (0.67-1.63), p=0.839 | 0.93 (0.62-1.4), p=0.726 |
|  | other histiotypes | 0.96 (0.78-1.19), p=0.739 | 0.97 (0.77-1.22), p=0.801 |
| cancer diameter [mm] | continuous | 1 (1-1), p=0.031 | - |
| T stage | T1 | 1 (reference) | 1 (reference) |
|  | T2 | 1.27 (0.98-1.66), p=0.073 | 1.38 (1.06-1.81), p=0.019 |
|  | T3 | 2 (1.57-2.56), p<0.001 | 2.15 (1.65-2.8), p<0.001 |
|  | T4 | 3.4 (2.59-4.47), p<0.001 | 2.72 (2.02-3.65), p<0.001 |
|  | TX | 2.64 (1.6-4.37), p<0.001 | 2.08 (1.35-3.21), p<0.001 |
| histological tumor necrosis | no necrosis | 1 (reference) | 1 (reference) |
|  | necrosis | 1.25 (1.01-1.56), p=0.045 | 1.42 (1.11-1.82), p=0.005 |
| synchronous tumor metastases | no metastases | 1 (reference) | 1 (reference) |
|  | distant metastases | 3.21 (2.74-3.76), p<0.001 | 2.52 (2.05-3.1), p<0.001 |
| renal sarcoma surgery | no surgical resection | 1 (reference) | 1 (reference) |
|  | surgical resection | 0.3 (0.24-0.37), p<0.001 | 0.39 (0.31-0.49), p<0.001 |
| systemic therapy | no systemic therapy | 1 (reference) | 1 (reference) |
|  | systemic therapy | 1.18 (1-1.38), p=0.044 | 0.68 (0.55-0.84), p<0.001 |
| regional lymph node surgery | No regional lymph node surgery | 1 (reference) | - |
|  | Regional lymph node surgery | 0.88 (0.75-1.04), p=0.145 | - |
|  | Unknown if there was any regional lymph node surgery | 1.61 (0.92-2.83), p=0.096 | - |
| resection margin | R0 | 1 (reference) | * |
|  | R+ | 1.78 (1.45-2.18), p<0.001 | - |
|  | RX | 1.12 (0.78-1.6), p=0.552 | - |
| facility type | academic/research program | 1 (reference) | - |
|  | other treatment facility type | 0.89 (0.76-1.03), p=0.113 | - |
| facility location | South Atlantic | 1 (reference) | - |
|  | East North Central | 1.05 (0.8-1.39), p=0.722 | - |
|  | East South Central | 0.94 (0.65-1.36), p=0.754 | - |
|  | facility location suppressed for age 0-39 years | 0.63 (0.46-0.85), p=0.002 | - |
|  | Middle Atlantic | 0.97 (0.75-1.26), p=0.831 | - |
|  | Mountain | 0.88 (0.56-1.38), p=0.57 | - |
|  | New England | 1.19 (0.81-1.76), p=0.365 | - |
|  | Pacific | 0.99 (0.74-1.32), p=0.924 | - |
|  | West North Central | 0.96 (0.74-1.25), p=0.752 | - |
|  | West South Central | 0.86 (0.6-1.23), p=0.419 | - |
| year of diagnosis | 2004-2010 | 1 (reference) | - |
|  | 2011-2016 | 1.24 (1.06-1.44), p=0.006 | - |

Supplemental table 5: multivariable weighted Cox proportional hazards model in patients receiving surgical resection of renal sarcomas and data on survival time and status (n=949).

| variable | level | multivariable HR |
| --- | --- | --- |
| age | continuous | 1.01 (1.01-1.02), p<0.001 |
| gender | male | 1 (reference) |
|  | female | 0.81 (0.67-0.97), p=0.026 |
| renal sarcoma histiotype | Leiomyosarcoma, NOS | 1 (reference) |
|  | Angiosarcoma | 2.91 (2.15-3.92), p<0.001 |
|  | Malignant rhabdoid tumor | 1.12 (0.81-1.54), p=0.499 |
|  | Dedifferentiated liposarcoma | 0.8 (0.48-1.31), p=0.375 |
|  | Primitive neuroectodermal tumor, NOS | 0.91 (0.59-1.39), p=0.652 |
|  | Ewing sarcoma | 1.05 (0.58-1.92), p=0.863 |
|  | Spindle cell sarcoma | 0.83 (0.45-1.54), p=0.563 |
|  | Clear cell sarcoma of kidney | 0.88 (0.52-1.48), p=0.627 |
|  | other histiotypes | 1.03 (0.8-1.34), p=0.797 |
| tumor metastases | no metastases reported | 1 (reference) |
|  | distant metastases | 2.24 (1.79-2.81), p<0.001 |
| T stage | T1 | 1 (reference) |
|  | T2 | 1.52 (1.09-2.12), p=0.013 |
|  | T3 | 2.65 (1.94-3.6), p<0.001 |
|  | T4 | 3.91 (2.73-5.6), p<0.001 |
|  | TX | 1.13 (0.49-2.59), p=0.776 |
| histological tumor necrosis | no necrosis | 1 (reference) |
|  | necrosis | 1.38 (1.07-1.78), p=0.014 |
| resection margin | R0 | 1 (reference) |
|  | R+ | 1.36 (1.1-1.68), p=0.004 |
|  | RX | 1.07 (0.72-1.58), p=0.755 |
| systemic therapy | no systemic therapy | 1 (reference) |
|  | systemic therapy | 0.83 (0.66-1.06), p=0.131 |


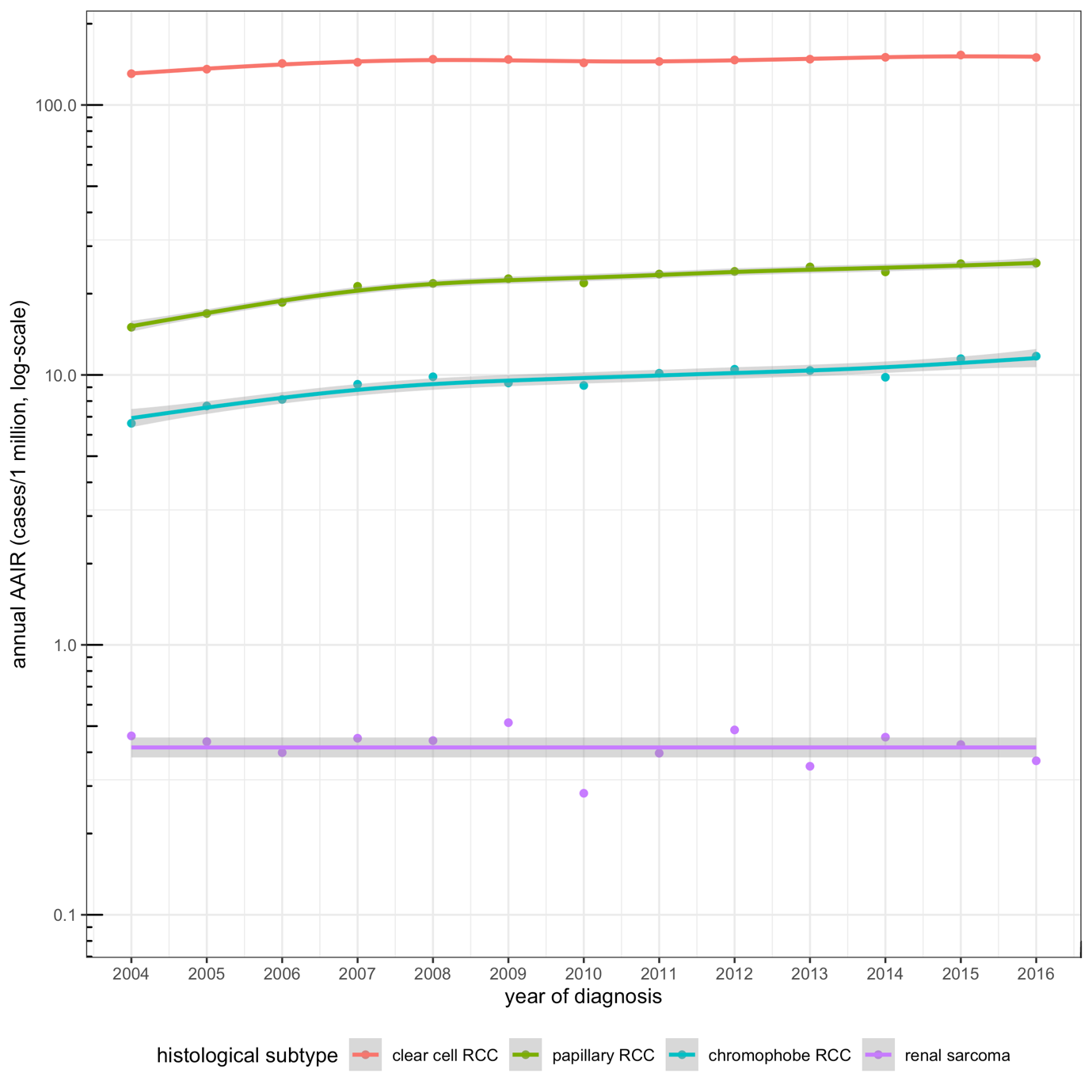


Supplemental figure 1: age-adjusted incidence rates of renal tumor subtypes over the years


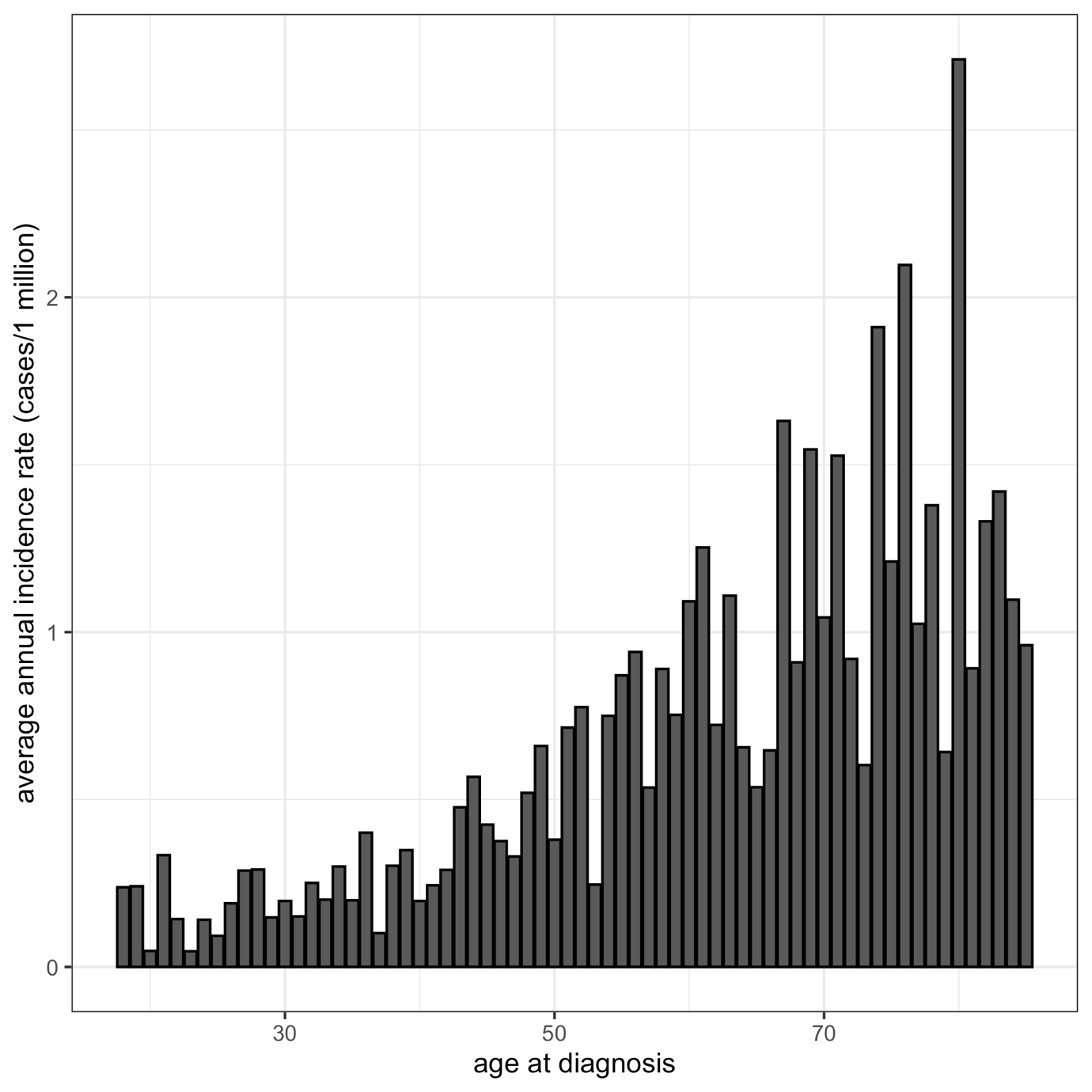


Supplemental figure 2: incidence rates of renal sarcomas according to age


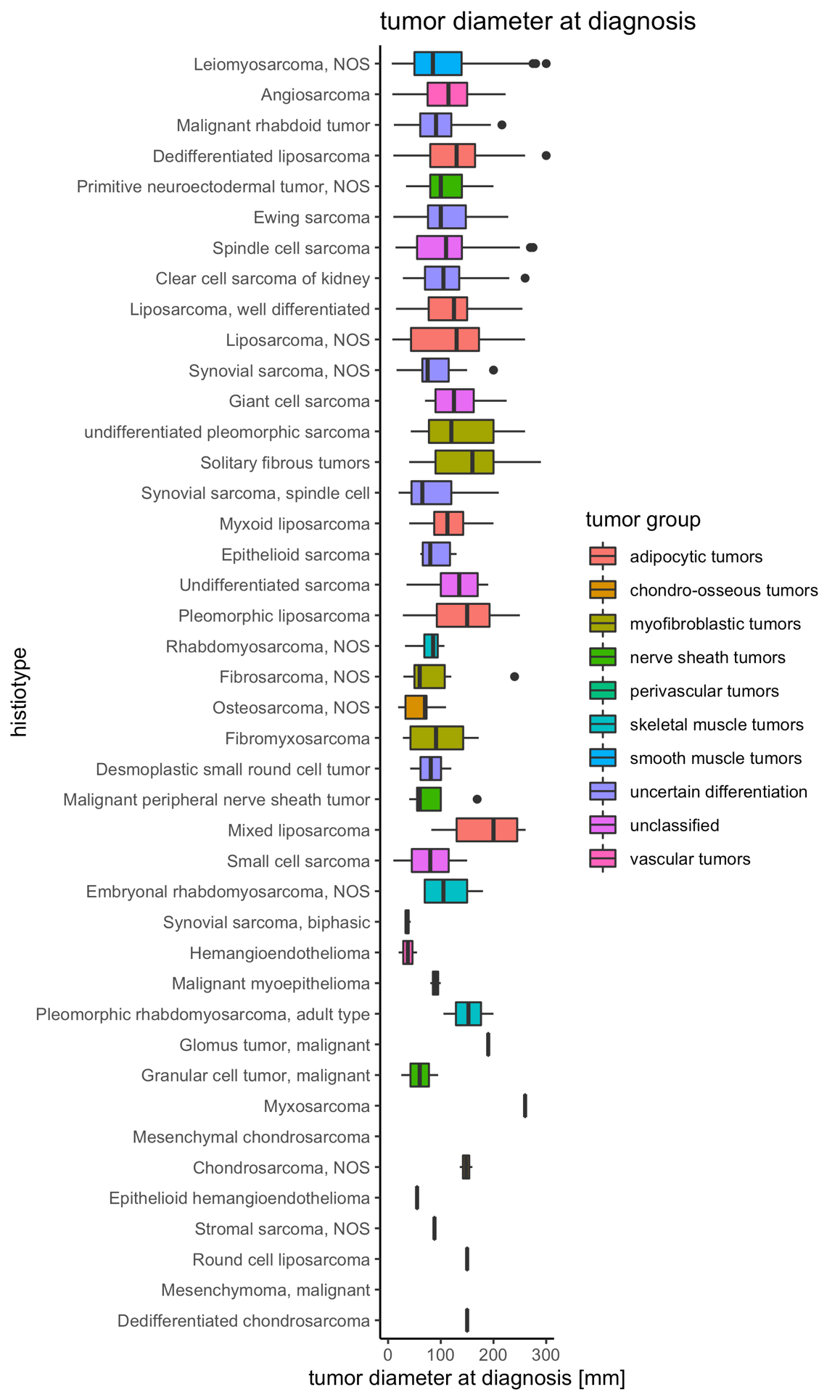


Supplemental figure 3: renal sarcoma diameter at time of diagnosis according to histiotype.


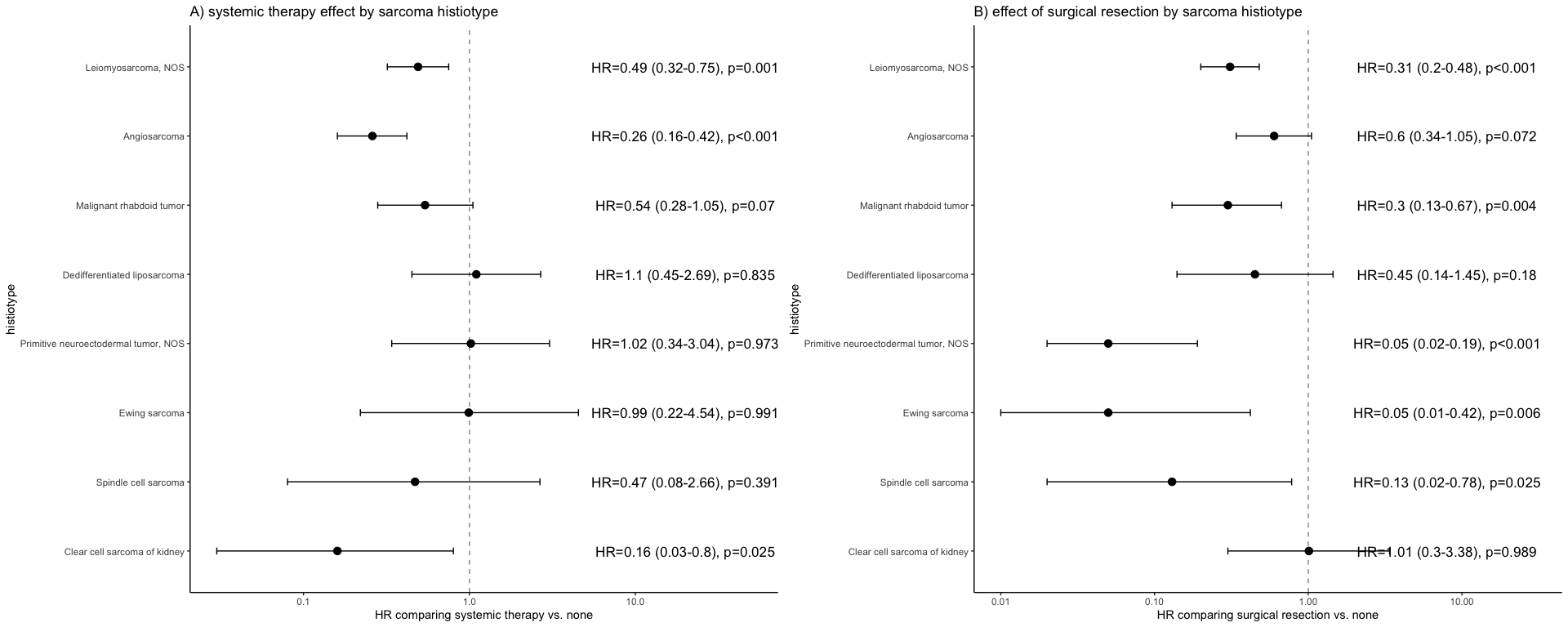


Supplemental figure 4: effect of A) systemic therapy vs. none and B) surgical resection vs. none stratified by sarcoma histiotype. Hazard ratios (HR) are provided after multivariable adjustment for age, gender, comorbidities, T stage, tumor necrosis, metastases and the respective treatment.
